# Advances in the Molecular Mechanisms of Abscisic Acid and Gibberellins Functions in Plants

**DOI:** 10.3390/ijms22116080

**Published:** 2021-06-04

**Authors:** Víctor Quesada

**Affiliations:** Instituto de Bioingeniería, Universidad Miguel Hernández, Campus de Elche, 03202 Elche, Spain; vquesada@umh.es; Tel.: +34-96-665-8812

In this special issue entitled, “*Advances in the Molecular Mechanisms of Abscisic Acid and Gibberellins Functions in Plants*”, eight articles are collected, with five reviews and three original research papers, which broadly cover different topics on the abscisic acid (ABA) field and, to a lesser extent, on gibberellins (GAs) research. These works explore ABA involvement in processes like flowering, plant defense, abiotic stress response or maturation of non-climacteric fruits, with reports in the last case of interplay between ABA and GAs. New findings on the regulation of ABA or GAs activity are also reported in this issue. The experimental studies and reviews published in this special issue focus on the results obtained using principally the plant model system *Arabidopsis thaliana*. Notwithstanding, other works based on agronomically important plants, such as grapevine or citrus species, are also included, and reveal the crucial role of ABA and GAs across different plant species. I summarize here the main findings of these works, which represent outstanding advances in our knowledge on the molecular mechanisms through which ABA and GAs control fundamental processes in plants.

Phytohormones GAs and ABA antagonistically regulate both plant growth and several developmental processes, such as seed maturation, seed dormancy, germination, hypocotyl elongation, primary root growth and flowering time. ABA and GAs generally inhibit and promote respectively cell elongation and growth, and the mutual antagonism between these two phytohormones governs many developmental decisions in plants [1].

In addition to the growing body of evidence for ABA as a modulator of plant growth and development [2], ABA is primarily known for being a fundamental player in the response, tolerance and adaptation of plants to diverse abiotic stress conditions, among which low temperatures, heat, drought, salinity or flooding are highlighted [3]. Interestingly, different recent works suggest a function for GAs in controlling some biological processes in response to stress [4,5,6].

This special issue “Advances in the Molecular Mechanisms of Abscisic Acid and Gibberellins Functions in Plants” contains eight articles; most of these focus on ABA, five are review articles and three are original research papers published by field experts. These manuscripts will help to understand the fundamental roles of GAs and ABA in the regulation of plant growth, development, and in responses to abiotic or biotic stresses. These articles will also shed light on the molecular mechanisms of ABA and GAs action in plants. The reviews published in this issue focus on the interaction between ABA and GAs in regulating non climacteric fruit development and maturation [7], current knowledge on the role of ABA in mediating mechanisms whereby grapevine deals with abiotic stresses [8], the analysis of the role of ABA in flowering transition [9], the mechanism by which the type 2C Protein Phosphatases (PP2C) gene transcription modulates ABA signaling [10], and the role of the Mediator complex on the ABA signaling pathway and abiotic stress response [11]. The original research articles investigate the causes and consequences of the kaolin-induced modulation of ABA biosynthesis in grapevines when faced with a water deficit [12], the involvement of ABA in plant immune responses [13], and the relation between GAs and Arabidopsis receptor-like cytoplasmic kinase (RLCK) VI_A2 [14]. In this editorial, I summarize the main findings of these eight insightful works.

Fruit development and maturation results from an intricate interplay of molecular and physiological processes, regulated by endogenous (hormones) and external (environment) factors. The paper by Alferez et al. [7] exhaustively reviews the interplay between ABA and GAs in regulating the development and maturation of non-climacteric fruits at the molecular level, in economically important fruits like grape berries, strawberries and citruses. Alferez et al. [7] also relate the interaction of ABA and GAs with ethylene and sugar signaling in modulating non-climacteric fruit development and maturation. They depict a time-course model in which GA levels lower, ABA levels rise and ethylene production remains steady, whereas ethylene perception increases as non-climacteric fruit maturation progresses. Notwithstanding, Alferez et al. [7] also highlight that although current knowledge clearly points out the crosstalk between ABA and GAs as a major factor that controls fruit maturation, fine details of this regulation are still not well-understood and warrant further research. Finally, the authors examine the increasing body of evidence about ABA, GAs and the genus Citrus, which reveal that this woody genus can be considered an emerging plant model system for non-climacteric maturation studies.

Climate change poses a threat to important agricultural regions in the world, such as Mediterranean-climate areas, where socio-economically relevant crops (e.g., grapevine) can be seriously threatened. Consequently, improvements in viticulture techniques are needed, and better knowledge of grapevine physiology under stress conditions is required to achieve this. Besides its socio-economic importance, *Vitis vinifera* is also a model species in drought-response research. Marusig and Tombesi [8] provide an exhaustive overview of current knowledge on the role of ABA in mediating mechanisms whereby grapevines cope with abiotic stresses. In line with this, these authors especially focus on the mechanisms of ABA biosynthesis and translocation, the role of this phytohormone in regulating stomata closure and carbohydrates mobilization in response to drought stress, as well as ABA involvement in salt stress. The results of all these works clearly demonstrate that in *Vitis vinifera*, ABA is a key hormone involved in regulating the mechanisms for coping with major threats caused by climate change. Marusig and Tombesi [8] also highlight some main issues that deserve further research in this field, which are fundamentally the understanding of the role that ABA plays in drought stress in relation to water stress severity, duration and frequency, the interaction of ABA regulation and carbohydrates under water stress, and a profounder knowledge of the ABA function in the salt stress response.

Drought escape (DE), which is accelerated flowering that plants undergo in the vegetative phase in response to a water deficit, is considered an adaptive strategy for survival in dry climates. Studying DE in Arabidopsis has revealed that ABA plays a prominent role in controlling floral transition. The review by Martignago et al. [9] thoroughly explores current knowledge on how and in what spatial context ABA signals can affect the intricate floral network. These authors review ABA signaling and its multiple connections with the photoperiodic pathway, as the DE process is highly intertwined with photoperiodic genes, and integrating drought stimuli into the floral network is mediated largely by ABA. Martignago et al. [9] emphasize the important role of the *GIGANTEA* (*GI*) gene in integrating ABA signaling into flowering. *GI* is a key flowering gene required for photoperiod perception and clock function, and it also emerges as the key driver of DE. The current model suggests that the transcription factors (TFs) activated by ABA would recruit GI in different genomic positions to regulate gene networks involved in the drought stress response. These authors also analyze the putative role of FD and FD-like TFs in the modulation of ABA responses through interactions with FLOWERING LOCUS T (FT) and FT-like proteins at the shoot apex. Finally, Martignago et al. [9] discuss how the progress made in Arabidopsis about ABA-flowering molecular interactions can be transferred to crops. Along these lines, they highlight the utility of studying DE traits in natural populations, and the importance of rice as the DE pathway is essentially conserved in this species.

ABA plays a pivotal role in controlling plant stomata closure in response to osmotic stress, which prevents water loss. However even under stressful conditions, stomatal apertures occur to uptake CO_2_ for photosynthesis. Consequently, ABA levels must be very fine-tuned regulated to maintain plant homeostasis, which is achieved by controlling this hormone’s biosynthesis, catabolism and signaling pathway. Jung et al. [10] comprehensively review how plants modulate the ABA signaling pathway by focusing on the transcriptional regulation of *PP2C* gene expression by ABA. They report that plant *PP2Cs* are a fundamental switch at the core of the ABA signaling network. Jung et al. [10] summarize the results hitherto published and demonstrate that ABA induces both repressors and activators of *PP2C* gene transcription to modulate ABA responses. These regulators can also affect the chromatin state by thereby regulating the transcription of ABA-responsive genes. Remarkably, this stress-induced chromatin remodeling state can be memorized, and even inherited, by the next generation of plants.

The Mediator is a conserved eukaryotic multiprotein complex that modulates the association between transcription factors and RNA-polymerase II to accurately regulate gene transcription. The functions of the Mediator complex in plant development processes and the biotic stress response have been extensively studied. However, its roles on the ABA signaling pathway and abiotic stress responses are still poorly known. Chong et al. [11] exhaustively summarize current knowledge on the regulatory roles of the Mediator complex on the ABA signaling pathway and in plant responses to three abiotic stresses: cold, high salinity and drought. These authors show that the Mediator complex is critical for plants to respond to abiotic stresses, and particularly focus on the participation of Mediator subunits MED16, MED18, MED25 and CDK8 in response to ABA and environmental cues. Chong et al. [11] report that Mediator subunits display multifunctional roles in salt and drought stress, and they discuss further potential research approaches needed to ascertain the role of the Mediator complex in regulating ABA and abiotic stress responses.

Kaolin is a natural clay used in some crops to alleviate the negative impact of extreme temperatures and/or drought on leaves and fruits, due to its light reflective properties. In line with this, a reduction in leaf ABA content associated with better leaf stomatal conductance induced by kaolin has been reported in grapevines. The research manuscript by Frioni et al. [12] explores the causes and consequences of not only the kaolin-induced modulation of ABA biosynthesis under progressive water shortage, but also the dynamic interactions between kaolin and ABA precursors violaxanthin (Vx), antheraxanthin (Ax), zeaxanthin (Zx) (representing the molecules involved in the xanthophyll (VAZ) cycle) and neoxanthin (Nx). Frioni et al. [12] report that kaolin, under water deficit, preserves leaf transpiration and reduces the accumulation of ABA in grapevine leaves by avoiding the deviation of the VAZ epoxidation/de-epoxidation cycle in the biosynthesis of Nx. Their findings contribute to explaining the mechanisms involved in the kaolin-induced protection of canopy functionality.

In addition to the aforementioned role of ABA in both controlling plant responses to abiotic stress and regulating plant developmental processes and growth, this hormone is also involved in responses to biotic stresses caused by a wide range of plant pathogens. ABA’s role in plant pathogen response is complex, given its interplay with key players in defense (e.g., salicylic acid (SA), jasmonic acid (JA) and ethylene (ET)), and also because ABA outcomes depend on the biology of the infective pathogen. Furthermore, the specific receptor of ABA, which activates the positive or negative ABA responses during immune responses, has to date remained unknown. The comprehensive study by García-Andrade et al. [13] unveils a non-redundant role in plant immunity for one of the 14 multigene ABA receptor family members, namely ABA receptor PYRABACTIN RESISTANCE 1 (PYR1). This research manuscript reveals that this receptor is crucial for modulating the cross-talk between the SA and ET signaling pathways in plant defense. The results of García-Andrade et al. [13] also demonstrate that ABA-activated SNF1-related protein kinases (SnRKs) subfamily 2 (SnRK2s) are fundamental components for plant resistance to pathogens.

Valkai et al. [14] investigate the biological function of the Arabidopsis *RLCK VI_A2* gene in the regulation of plant growth and skotomorphogenesis. RLCK VI_A2 is a member of the plant-specific receptor-like cytoplasmic kinases (RLCKs) that form a large and barely characterized family. The activity of the RLCK VI_A class of dicots is regulated by Rho-of-plants (ROP) GTPases. Valkai et al. [14] show that loss of the *RLCK VI_A2* function leads to reduced cell expansion and seedling growth. These mutant phenotypes can be rescued by the exogenous application of GAs. However, differences in neither GA content nor GA sensitivity of the *RLCK VI_A2* defective mutant have been found compared to the wild type. An RNA-seq analysis indicated that the RLCK VI_A2 kinase and GAs can act in parallel to regulate cell expansion and plant growth. Interestingly, the transcriptomic analysis also revealed a role for RLCK VI_A2 kinase in cellular transport and cell wall organization.

Altogether, the contributions published in this special issue are excellent examples of the recent advances made in the molecular mechanisms of ABA and GA functions in plants. I wish to thank all the authors for their contributions and the reviewers for their critical assessments of these articles. I also thank the assistant editor Ms. Reyna Li for providing me with the opportunity to serve as the Guest Editor of this special issue.

## References

[1] Shu K., Zhou W., Chen F., Luo X., Yang W. (2018). Abscisic Acid and Gibberellins Antagonistically Mediate Plant Development and Abiotic Stress Responses. Front Plant Sci..

[2] Zhu J.K. (2002). Salt and drought stress signal transduction in plants. Annu. Rev. Plant Biol..

[3] Yoshida T., Christmann A., Yamaguchi-Shinozaki K., Grill E., Fernie A.R. (2019). Revisiting the Basal Role of ABA—Roles Outside of Stress. Trends Plant Sci..

[4] Hamayun M., Hussain A., Khan S.A., Kim H.Y., Khan A.L., Waqas M., Irshad M., Iqbal A., Rehman G., Jan S. (2017). Gibberellins producing endophytic fungus Porostereum spadiceum AGH786 rescues growth of salt affected soybean. Front. Microbiol..

[5] Urano K., Maruyama K., Jikumaru Y., Kamiya Y., Yamaguchi-Shinozaki K., Shinozaki K. (2017). Analysis of plant hormone profiles in response to moderate dehydration stress. Plant J..

[6] Wang B., Wei H., Xue Z., Zhang W.H. (2017). Gibberellins regulate iron deficiency-response by influencing iron transport and translocation in rice seedlings (*Oryza sativa*). Ann. Bot..

[7] Alferez F., Uilian de Carvalho D., Boakye D. (2021). Interplay between Abscisic Acid and Gibberellins, as Related to Ethylene and Sugars, in Regulating Maturation of Non-Climacteric Fruit. Int. J. Mol. Sci..

[8] Marusig D., Tombesi S. (2020). Abscisic Acid Mediates Drought and Salt Stress Responses in *Vitis vinifera*—A Review. Int. J. Mol. Sci..

[9] Martignago D., Siemiatkowska B., Lombardi A., Conti L. (2020). Abscisic Acid and Flowering Regulation: Many Targets, Different Places. Int. J. Mol. Sci..

[10] Jung C., Nguyen N.H., Cheong J.J. (2020). Transcriptional Regulation of Protein Phosphatase 2C Genes to Modulate Abscisic Acid Signaling. Int. J. Mol. Sci..

[11] Chong L., Guo P., Zhu Y. (2020). Mediator Complex: A Pivotal Regulator of ABA Signaling Pathway and Abiotic Stress Response in Plants. Int. J. Mol. Sci..

[12] Frioni T., Tombesi S., Sabbatini P., Squeri C., Rodas N.L., Palliotti A., Poni S. (2020). Kaolin Reduces ABA Biosynthesis through the Inhibition of Neoxanthin Synthesis in Grapevines under Water Deficit. Int. J. Mol. Sci..

[13] García-Andrade J., González B., González-Guzman M., Rodriguez P.L., Vera P. (2020). The Role of ABA in Plant Immunity is Mediated through the PYR1 Receptor. Int. J. Mol. Sci..

[14] Valkai I., Kénesi E., Domonkos I., Ayaydin F., Tarkowská D., Strnad M., Faragó A., Bodai L., Fehér A. (2020). The Arabidopsis RLCK VI_A2 Kinase Controls Seedling and Plant Growth in Parallel with Gibberellin. Int. J. Mol. Sci..

